# Cervical Thymic Cyst: A Rare Differential Diagnosis in Lateral Neck Swelling

**DOI:** 10.1155/2013/350502

**Published:** 2013-02-07

**Authors:** Vijendra Shenoy, M. Panduranga Kamath, Mahesh Chandra Hegde, Raghavendra Rao Aroor, Vijetha V. Maller

**Affiliations:** ^1^Department of ENT-Head and Neck Surgery, Kasturba Medical College-Mangalore, Manipal University, Mangalore, Karnataka 576 104, India; ^2^Department of Otolaryngology, Kasturba Medical College Hospital, Manipal University, Attavar, Mangalore, Karnataka 575 001, India; ^3^Department of Radiodiagnosis & Imaging, Kasturba Medical College-Mangalore, Manipal University, Mangalore, Karnataka 576 104, India

## Abstract

*Introduction*. Thymic cysts are among the rarest cysts found in the neck. Nests of thymic tissue may be found anywhere along the descent of the thymic primordia from the angle of the mandible to the mediastinum. Mediastinal extension is seen in 50% of cervical thymic cysts. *Case Report*. We report an uncommon case of a 15-year-old male, who noted a painless, growing mass on left side of his neck of one-year duration. Computerised tomographic scan showed a multiloculated fluid density lesion with enhancing septae in the left parapharyngeal space, extending from the level of mandible up to C7 vertebral level. Here, we discuss the surgical aspect, histopathology, and management of this rare lateral neck swelling. *Discussion*. Clinically, in most cases, cervical thymic lesions present as a unilateral asymptomatic neck mass, commonly on the left side of the neck, and 75% of patients present before 20 years of age. *Conclusion*. Thymic cyst should be included as differential diagnosis of cystic neck masses. Greater awareness among the pathologists may decrease misdiagnosis.

## 1. Introduction

Cervical thymic cysts are unusual lesions usually presenting in the 1st decade of life [1]. Two varieties of thymic cysts have been described, thymopharyngeal duct cysts and cysts arising from degeneration of Hassall's corpuscles within ectopic thymic remnants [2]. The presence of thymic tissue differentiates thymic cysts from 3rd and 4th branchial cysts [3].

We report a rare case of thymopharyngeal duct cyst in a 15-year-old male, describing CT findings, intraoperative findings and histopathology.

## 2. Case Report

A 15-year-old boy presented to our outpatient department with a swelling over the left side of neck of 1-year duration which had gradually increased in size over time with no associated pain, weight loss, or pressure symptoms. There was no significant family history. His general physical examination appeared normal without any obvious deformities or abnormalities. Local examination revealed a 3 × 2 cm oval, nontender, and firm mass on the left side of neck at level III. All his other systems seemed to be normal clinically. 

**Figure 1:**
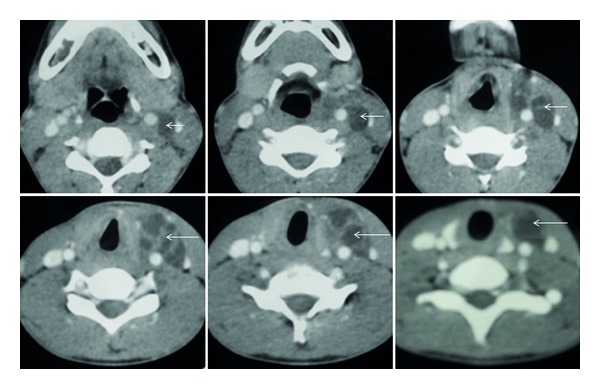
CT scan of the neck showed a multiloculated fluid density lesion with enhancing septae in the left parapharyngeal space, extending from the level of mandible up to C7 vertebral level.

CT scan of the neck (Figure 1) showed a multiloculated fluid density lesion with enhancing septae in the left parapharyngeal space, extending from the level of mandible up to C7 vertebral level. The mass was extending posteromedially to the left sternocleidomastoid muscle extending from the level of the thyroid cartilage up to the level of the superior mediastinum. Fine needle aspiration cytology of the swelling was suggestive of an infected branchial cyst. Thyroid function tests were normal. All the haematological and biochemical investigations were within normal range.

**Figure 2:**
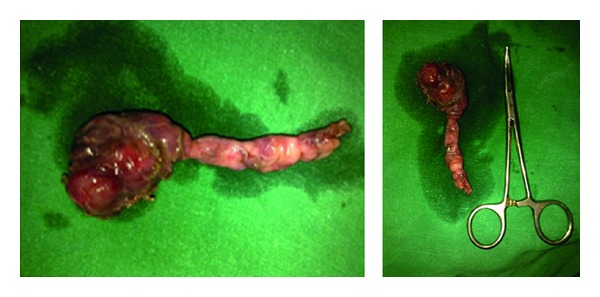
Surgical specimen showing cyst with fibrous cord tracking inferiorly up to the superior mediastinum.

The mass was excised under general anaesthesia with a horizontal incision two finger breadths below the body of the mandible. Surgical exploration revealed that a cystic mass surrounded by fibrous adhesions was found lying between the left lobe of thyroid gland medially, internal jugular vein laterally, and the sternocleidomastoid anterolaterally extending 1 cm below the angle of the mandible to the level of the cricoid cartilage. The cyst was found to have a fibrous cord tracking inferiorly up to the superior mediastinum, for which a stepladder incision was made 2 cm inferior to the upper incision, and the cord was dissected and ligated deep to the clavicle. The mass was completely excised (Figure 2).

**Figure 3:**
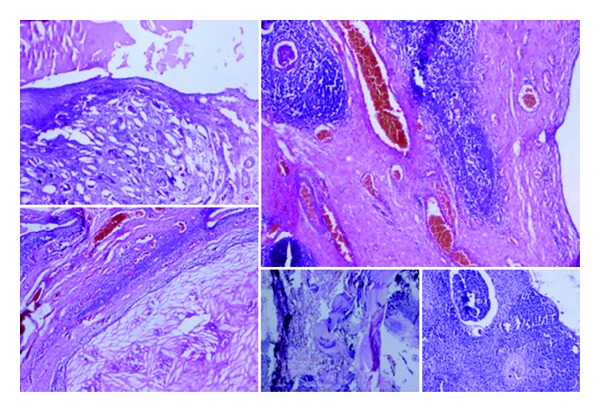
Histopathological examination revealed multiple cystic spaces lined by cuboidal to squamous epithelium. The cyst contained numerous cholesterol clefts with subepithelium showing fibrocartilagenous tissue, foreign body corpuscles, and Hassall's corpuscles.

 Histopathological examination revealed multiple cystic spaces lined by cuboidal to squamous epithelium. The cyst contained numerous cholesterol clefts with subepithelium showing fibrocartilagenous tissue, foreign body corpuscles, and Hassall's corpuscles thereby diagnosed as a thymic cyst (Figure 3).

A final diagnosis of thymic cyst was made. Postoperative period was uneventful. The patient has been symptom free for more than a year.

## 3. Discussion

Differential diagnosis for cystic masses in the neck is done. Cystic masses of the neck are thyroglossal duct cysts, branchial cleft cysts, cystic hygromas, dermoid cysts, epidermoid cysts, thymic cysts, bronchogenic cysts (visceral cysts), and laryngoceles. Thymic cysts are very rare representing only 1% of cystic cervical masses [1]. A study conducted in 20 years by Hsieh et al. on 331 patients under the age of 18 years presenting with cystic neck masses found that 181 (54.68%) patients had thyroglossal cysts, followed by cystic hygromas (83 patients, 25.08%), branchial cleft cysts (54 patients, 16.31%), and bronchogenic cysts (3 patients, 0.91%), and nine cases (2.72%) remained unclassified. Only one case was diagnosed as thymic cyst (0.30%) [4].

Patients with thymic cysts usually present in the 1st decade of life being more common in males. Patients usually present with slow growing painless mass in the lateral aspect of neck near thoracic inlet either deep or superficial to sternocleidomastoid muscle. The size can vary from 1 cm to 26 cms [2]. Huge cervical thymic cysts can compress the neighbouring structures leading to dysphagia, dyspnoea, and hoarseness of voice [1].

Two varieties of thymic cysts are described, congenital and acquired. Persistence of thymopharyngeal tracts and the degeneration of Hassall's corpuscles within ectopic thymic remnants are the two most important etiologies of thymic cysts [5]. The thymopharyngeal duct arises from the developing pyriform sinus and descends into the mediastinum, traveling lateral to the thyroid gland. Thymic tissue nests can be found along the path of descent of the thymic primordia from the angle of the mandible to the mediastinum [3]. Hence, cervical thymic cysts can be found anywhere from the angle of the mandible to the thoracic inlet, most commonly on lateral aspect on left side [3]. Thymopharyngeal duct cysts are rarer variety of thymic cysts [6].

The cervical thymic lesions include the following categories distinguished by anatomic location and the nature of the thymic gland tissue.

*Accessory Cervical Thymus. *Solid cervical thymic tissue is sequestered from the main gland, along the normal descent path, with or without parathyroid. Previous terms include aberrant, ectopic, undescended, persistent, or accessory thymus.

*Cervical Thymic Cyst. *Sequestered cystic cervical thymus is found along a normal path of descent, with or without parathyroid glands. It is a cystic version of accessory cervical thymus and may have fibrous band or a solid thymic cord connection to the pharynx or mediastinum.

*Undescended Cervical Thymus. *This occurs when a solid lobe of thymus fails to descend entirely, with or without a parathyroid complex. It differs from accessory cervical thymus in that only half of the normally blobbed thymus is present in the mediastinum; conceivably, it may also become cystic.

*Persistent Thymopharyngeal Duct Cyst. *This is the same as undescended cervical thymus; however, the thymic duct is cystic. The thymus is solid, with or without parathyroid complex, and probably represents undescended thymus. A variant would be the cervical cystic duct leading to the mediastinal thymus.

*Persistent Thymic Cord. *This is the cervical prolongation of a solid thymic cord which is continuous with the mediastinal thymus. The cystic variant may overlap with the histology and clinical appearance of the cervical thymic cyst if a true connection to mediastinal thymus cannot be documented.

*Cervical Extension of Mediastinal Thymus. *This appears as low midline solid thymus at the thoracic inlet due to incomplete mediastinal descent. It may transiently present with increased intrathoracic pressure.

*Ectopic Thymus. *This is the rare, solid thymic tissue in abnormal locations, for example, in the pharynx, trachea, or base of skull [7, 8].

On CT, thymic cysts usually have a homogeneous low attenuation (10 to 20 HU) with a thin uniformly smooth wall. Thymic cysts may be unilocular or multilocular. After contrast injection the cyst wall shows smooth regular enhancement. When a cyst becomes infected, its protein content increases, and this is seen on CT as an increase in attenuation. On MR imaging, thymic cysts are homogeneous with a low or intermediate signal on T1-weighted and a high signal on T2-weighted images. Relationship of the cyst with surrounding structures can be clearly demonstrated on CT and MRI.

Thymic cysts are unilocular or multilocular containing brownish fluid. The cyst wall lining ranges from flattened squamous or cuboidal cells to multilayered stratified squamous epithelium to even primitive respiratory epithelium. Lobulated lymphoid tissue in the cyst wall contains Hassall's corpuscles [3]. The increasing number of cervical thymic cysts reported in the last few years probably reflects greater awareness of this condition among pathologists. It is also possible that in the past, many cases of thymic cyst had been missed and diagnosed as brachial cleft cyst because of inadequate sampling of the specimen. The frequent atrophic condition of the thymic remnants may require sampling from various portions of specimen before a diagnosis of thymic cyst could be rendered [9]. 

We report this case to highlight the importance of thymic cyst as a differential diagnosis of cystic lateral neck mass, although it is very rare. Also, in our case, cyst was found to have a fibrous cord tracking inferiorly up to the superior mediastinum; hence, we believe it could have arisen from remnant thymopharyngeal duct, which is rarer variety of thymic cyst in the neck.

## 4. Conclusion

 Thymic cysts are unusual causes of cystic cervical masses. However, they should be included as differential diagnosis of cystic neck masses. Imaging, surgical findings, and histopathological correlation play an important role in diagnosing thymic cysts. This paper could be summarized as follows. Thymic cyst is a very rare differential diagnosis of cystic lateral neck swelling. According to the various literatures published, thymic cyst accounts for less than 0.5% of cystic neck swelling. Mediastinal extension is seen in 50% of cervical thymic cysts. Thymic cyst is thought to develop from persistent thymopharyngeal tracts and the degeneration of Hassall's corpuscles within ectopic thymic remnants.
